# Medical News and Miscellany

**Published:** 1891-01

**Authors:** 


### Medical News and Miscellany.

---

The Medical Mirror, ($2) and DANIEL'S TEXAS MEDICAL JOURNAL, ($2) both for $3 for 1891.

---

The New Orleans Medical Journal, ($3) and this journal, ($2) both for $4. Subscribe at once.

---

Dr. E. G. Cochran has been appointed assistant surgeon of the Mexican Central R. W. system at Silao, Mexico, vice Dr. A. S. Paul, resigned.

**We are Authorized** to say that Dr. E. J. Beall, of Fort Worth, Texas, expects shortly to receive a supply of the "Koch Lymph," and desires to correspond with physicians in Texas who meet with cases of Lupus; looking to undertaking the treatment by the new method.

---

**Dr. A. S. Wolff**, the quarantine officer of Brazos Santiago, has recently been ordered by his chief, State health officer Rutherford, to Claude and Vernon, near the Colorado line, where the small-pox had broken out. He soon got it under control, and vaccinated all the unprotected.

---

**Dr. E. J. Ward** has removed from Waxahachie to Corpus Christi, the change being induced by the state of his health. He hopes to find the sea air beneficial, and went to Corpus Christi originally for his health. Dr. Worsham succeeds to the practice of Ward & Worsham, at Waxahachie.

---

**The New State Health Officer of Texas.**—Governor Hogg in response to numerous petitions from all parts of the State, and by the business men of cities as well as leading physicians, has appointed Dr. R. M. Swearingen State Health Officer for the ensuing term. This meets with very general favor.

---

**Removals.**—Dr. W. T. Strain has removed from McCoy city to Wills Point.

Dr. J. M. Dollar has removed from Gauze to Burlington, same county, Milam.

Dr. E. S. Adams has removed from Caledonia to Garrison.

---

**An Austin** physician suggests for gonorrhœa subacute:

℞ Salol, gr. iiss.;

Terpin. hydrate, gr. iiss.

M. ft. capsule i.

Sig. One dose, repeat every 4 hours.

For Sale.—I will sell my residence and drugstore with good practice. Apply to S. H. HARDY, M. D.,
Paige, Texas.

[This is in a good agricultural section and presents a rare opportunity to step into a good practice.—ED.]

---

Small-pox.—Austin had one case. It died to-day, Jan. 14. Holland has a few cases, but they have been isolated and the population vaccinated. The same at Temple and other places in the State. There is no ground for apprehending an epidemic at any point in the State. As soon as a case is reported, it is at once isolated and the people vaccinated, which puts an end to the spread of the disease at that point.

---

The University Medical Magazine.—It is announced that the size of this excellent journal is soon to be increased by the addition of from sixteen to twenty-four pages to each number, mainly to give space for fuller abstracts of current literature under the direction of Dr. Wm. Pepper and Dr. James Tyson (medicine), Dr. D. Hayes Agnew and Dr. J. William White (surgery), Dr. Horatio C. Wood (therapeutics), Dr. William Goodell (gynecology), and Dr. Barton C. Hirst (obstetrics).

---

The Committee on Medical Legislation of the Texas State Medical Association have drafted suitable bills, one to regulate the practice of medicine, and one creating a State Board of Health; and will present them to the Legislature early in the present session. Backed as they are by a string of petitions from the people, there is every reason to believe they will be passed, of course with more or less alteration. The next issue of the JOURNAL will contain the full text of both bills.

---

The Journal regrets exceedingly to chronicle the death of Dr. Wm. A. McGehee. He died just before Christmas, of dysentery, at Dripping Springs, Hays county, where he had only recently located. He practiced at Uvalde in 1886, removed to Alpine in 1888 or 1889, and thence last year to Hays county. He leaves a wife and five children. He was a brother of Senator George McGehee, we believe, and was a man very generally esteemed by those who knew him, and a conscientious practitioner of the healing art, to which he was devoted.

---

**The Great Secret.**—At last it transpires that "Koch's cure" is made public. What a spectacle has been presented all this time, in the medical profession using a remedy, the composition of which has been unknown to them! The Code prohibits the use of secret nostrums, and yet, what could Koch's lymph be called all this time, if *not* a nostrum? As predicted by the JOURNAL, it turns out to be a gelatine culture of the tubercle baccilli. Dr. Hamilton, Surgeon-General, M. H. S., predicts that the United States government will appropriate $10,000 to establish a factory for the production of the lymph.

---

Dr. H. K. Leake, of Dallas, who for many years has given special attention to gynecology, and who has written some of the best papers on allied subjects that have ever been read before the Association, is having erected in Dallas an institution for the reception and treatment of gynecological cases, on the plan adopted by Mr. Tait. Last summer Dr. Leake spent some months with Mr. Tait, as a private student, and received a thorough and minute instruction in all of his *technique*. When the building is completed Dr. Leake will announce the fact, and will receive a limited number of patients for treatment. We bespeak for him a generous support from the profession.

